# Transcriptomic and proteomic profiling of early blood responses reveals pathways associated with distinct clinical outcomes following H7N1 high pathogenicity avian influenza virus infection in chickens

**DOI:** 10.1016/j.psj.2026.107527

**Published:** 2026-08-06

**Authors:** María J Valdez-May, Albert Perlas, Miquel Nofrarías, Sonia Pina-Pedrero, Marc Dabad, Rosa Valle, Marta Pérez, Alejandro Moreno-León, Anna Esteve-Codina, Jordi Argilaguet, Kateri Bertran, Natàlia Majó

**Affiliations:** a Unitat mixta d’Investigació IRTA-UAB en Sanitat Animal. Centre de Recerca en Sanitat Animal (CReSA). Campus de la Universitat Autònoma de Barcelona (UAB), Bellaterra, 08193, Catalonia, Spain; b IRTA. Programa de Sanitat Animal. Centre de Recerca en Sanitat Animal (CReSA). Campus de la Universitat Autònoma de Barcelona (UAB), Bellaterra, 08193, Catalonia, Spain; c Universitat de Barcelona (UB), Barcelona, Spain; d Centre Nacional d'Anàlisi Genòmica (CNAG), Baldiri Reixac 4, 08028 Barcelona, Spain; e Departament de Sanitat i Anatomia Animals. Facultat de Veterinària. Campus de la Universitat Autònoma de Barcelona (UAB), Bellaterra, 08193, Catalonia, Spain

**Keywords:** HPAIV, Resilience, transcriptomics, proteomics

## Abstract

High pathogenicity avian influenza (HPAI) poses a significant threat to poultry. Some chickens show resilience to HPAI, but the mechanisms involved are poorly understood. In this study, we aimed to identify the biological mechanisms associated with resilience to HPAIV infection in chickens. Chickens were inoculated with H7N1 HPAIV and classified as susceptible or resilient based on clinical signs, mortality, histopathological lesions, AIV antigen detection in tissues, and viral shedding. Blood transcriptomic analysis revealed that genes from resilient chickens are involved in the integrin-mediated signaling pathway (*ITGA5, ITGA6, ITGB1*), as well as in adaptive (*BTK, TEC*) and innate (*TRIM13, LCK, TICAM1*) immune pathways. Plasma proteomic profiling revealed that ARF4, EIF4A2, and HNRNPAB (proteins involved in fundamental cellular processes) were less abundant in resilient chickens compared to both controls and susceptible birds. Our results suggest that early modulation of these pathways is associated with resilience to HPAIV and likely reflects early viral control or reduced systemic dissemination rather than the absence of infection.

## Introduction

Avian influenza (AI) is a viral disease with significant economic consequences for the global poultry industry, wildlife conservation, and public health. It is caused by avian influenza virus (AIV), which is a negative-sense, single-stranded RNA (ssRNA) virus belonging to the Orthomyxoviridae family (ICTV et al., 2026^1^). AIV evolves rapidly through random mutations and genetic reassortment (Swayne and Suarez, 2000). AIVs are classified as low pathogenicity (LPAIVs) or high pathogenicity (HPAIVs) based on molecular features and their ability to cause disease in chickens. LPAIVs are generally subclinical or lead to mild clinical signs. In contrast, HPAIVs induce acute, severe systemic disease and are linked to high mortality rates in chickens (Swayne and Suarez, 2000).

Understanding the mechanisms that determine resilience or susceptibility to HPAIV infection has been the focus of study for many research groups (Chen et al., 2025). Host responses to AIV differ between avian species (Matsuu et al., 2016). For example, the lack of the *RIGI* gene in chickens, unlike in ducks, has been suggested as a key factor contributing to their higher susceptibility to HPAIV (Barber et al., 2010). The response to AIV may vary not only between breeds but also among individuals within the same species (Matsuu et al., 2016). In this regard, the lung transcriptomic profile of the resilient Fayoumi indigenous chicken breed revealed pathways associated with the immune response, oxygen transport and circulation, and cell adhesion molecules when compared to the susceptible Leghorn chicken breed (Wang et al., 2014). Furthermore, commercial chickens that survived the H5N2 HPAI outbreaks in Iowa, USA, in 2015 exhibited single-nucleotide polymorphisms in genes such as TNF (tumor necrosis factor), IFNK (interferon kappa), and SPPL2B (signal peptide peptidase-like 2B), which are involved in inflammation, apoptosis, antiviral and adaptive immune responses, and B- and T-cell regulation, respectively (Drobik-Czwarno et al., 2018). Finally, in our previous study on lung gene expression in H7N1 HPAIV-resilient chickens, we observed downregulation of genes belonging to NF-κB pathways. Notably, the serine protease-encoding *PLAU* gene emerged as a key candidate, suggesting that early modulation of these pathways and genes may influence the clinical outcome of HPAIV infection (Perlas et al., 2021). More recently, we extended these findings to the upper respiratory tract, identifying early transcriptomic signatures in the nasal turbinates and lungs, particularly involving MAPK signaling, immune regulation, and antiviral pathways, that distinguish resilient from susceptible chickens (Valdez-May et al., 2026).

Despite extensive efforts, the mechanisms defining resilience and susceptibility to HPAIV infection in chickens remain poorly understood (Vu et al., 2022; Bryson et al., 2023). Clarifying how immune responses and genetic factors shape resilience to HPAIV could be key to finding new control strategies. Identifying genes that could be edited or proteins that serve as reliable biomarkers would be a crucial step toward breeding chickens better equipped to withstand influenza infections. In this study, we aimed to understand the biological mechanisms and identify potential pathways linked to the molecular basis of resilience or susceptibility in commercial broiler chickens at the early stages after experimental HPAIV infection using blood samples.

## Materials and methods

### Virus

The HPAIV strain A/Chicken/Italy/5093/1999 (H7N1) was used as the challenge virus. This H7N1 HPAIV strain has been previously used by our group (Sánchez-González et al., 2020; Valdez-May et al., 2026), where it induces approximately 50% mortality in chickens when administered intranasally at 10⁵ mean egg lethal doses (ELD_50_). This model enables the comparison of birds with divergent clinical outcomes, including resilient and susceptible phenotypes.

### Animals, housing, and experimental design

Animals, housing, conditions, and experimental procedures were as previously described (Valdez-May et al., 2026). Briefly, ninety Ross 308 commercial broiler chickens (*Gallus gallus domesticus*) were obtained from a commercial producer in Catalonia, Spain. Embryonated chicken eggs were incubated in a commercial hatchery, and, after hatching, chicks were vaccinated with the live attenuated Nobilis® Ma5 against the infectious bronchitis virus. Birds were housed in biosafety level 3 (BSL-3) facilities at IRTA-CReSA under negative-pressure HEPA-filtered conditions with ad libitum access to feed and water.

At 15 days of age, 10 chickens were used as negative controls and 80 chickens were intranasally inoculated with 10^5^ ELD_50_ of H7N1 HPAIV in 0.05 ml. Birds were monitored daily using a standardized scoring system (WOAH, 2024)^2^ and euthanized upon reaching endpoint criteria. Blood samples, oropharyngeal (OP) swabs, and cloacal (CL) swabs were collected at 8, 24, 48, and 72 hours post-inoculation (hpi). Necropsy and tissue collection for histopathology and immunohistochemistry were performed as previously described (Valdez-May et al., 2026).

### Quantitative PCR for the detection of HPAIV

RNA from OP and CL swabs was extracted using the IndiMag®Pathogen Kit (INDICAL Bioscience, Leipzig, Germany) in combination with the KingFisher Sample Purification System (Thermo Fisher Scientific, Waltham, MA, USA), according to the manufacturer’s instructions. In addition, total RNA was isolated from whole-blood samples using a phenol-based method. Purification was carried out with the RNeasy Mini Kit (Qiagen, Hilden, Germany) following the manufacturer's guidelines, including a 15-min treatment at room temperature with RNase-Free DNase Set (Qiagen). Viral RNA levels were quantified by TaqMan RT-qPCR using the 7500 Fast Real-Time PCR System (Thermo Fisher Scientific) (Heine et al., 2007).

### Classification of chickens as resilient or susceptible

Chickens were classified according to clinical and pathological outcomes after H7N1 HPAIV infection. Then, birds were classified as susceptible if they exhibited all of the following: (i) clinical signs such as depression, prostration, and/or dyspnea; (ii) histopathological lesions including inflammation, hemorrhages, and/or tissue necrosis; (iii) the presence of nucleoprotein antigen in tissues with histopathological lesions; and (iv) viral RNA detection (Ct < 35) from OP and/or CL swabs at 48 and/or 72 hpi. Birds were classified as resilient when they: (i) showed no clinical signs; (ii) had no detectable AIV nucleoprotein antigen by IHC; (iii) had no histopathological lesions consistent with HPAIV infection; and (iv) had either no detectable viral RNA or only low viral RNA levels, defined as Ct values >35 in OP and/or CL swabs. Since clinical signs and mortality appeared at 48 hpi, the classification was conducted from this time point onward.

In this study, the term “resilient” refers to birds showing a favorable clinical and pathological course of the infection. This classification does not imply the absence of infection or inherent resistance to HPAIV, as viral replication may occur below the detection limit or may be restricted to tissues not analyzed by RT-qPCR in this study.

### Host transcriptome analysis (RNA-Seq) on blood samples

RNA-seq experimental procedures and data analysis were conducted as previously described (Valdez-May et al., 2026). In brief, total RNA was extracted from whole-blood samples collected at 48 hpi and quality-checked (RIN > 9) before library preparation and sequencing at the National Center for Genomic Analysis (CNAG, Barcelona, Spain). Samples were excluded if they were unavailable, had insufficient volume, failed to meet RNA quality requirements, or were identified as transcriptomic quality-control outliers. The indeterminate infected bird was also excluded from comparative analyses. Libraries were prepared using the KAPA Stranded mRNA-Seq Kit for Illumina Platforms (Roche, Basel, Switzerland) and sequenced on an Illumina NovaSeq 6000 platform (paired-end, 2 × 51 bp).

Reads were aligned to a combined *Gallus gallus* (GRCg7b) and H7N1 HPAIV genome (GenBank DQ991317–DQ991332) using STAR v2.7.8, and gene quantification was performed with RSEM v1.3.0 (Li and Dewey, 2011). Differential expression was analyzed with limma v3.42.3, after TMM normalization and voom transformation, including group and sex as fixed effects. An empirical Bayes approach was used for variance moderation, and *P*-values were adjusted using the Benjamini–Hochberg procedure. Genes with FDR < 0.05 were considered differentially expressed. Principal component analysis (PCA) was performed on the voom-transformed log2 counts-per-million matrix using the top 500 most variable genes to assess sample structure and identify potential outliers. Principal components were evaluated to determine separation according to phenotype (HPAIV-resilient, HPAIV-susceptible, control).

Functional enrichment analysis was conducted using DAVID Bioinformatics Resources v6.8 (accessed July 8, 2026). DEG lists were uploaded as Ensembl gene identifiers and analyzed using *Gallus gallus* as the reference species. The background gene list consisted of all genes retained after low-expression filtering in the RNA-seq analysis, defined as genes with counts per million (CPM) > 1 in at least two samples. This background gene list was uploaded to DAVID and used for enrichment testing. For the susceptible and resilient DEG sets, enrichment analyses were performed using genes meeting the predefined differential expression threshold of |logFC| ≥ 1.5. For DEGs uniquely found in resilient birds, an exploratory analysis was performed using all DEGs regardless of fold-change magnitude. Upregulated and downregulated genes were analyzed separately. Gene Ontology (GO) Biological Process terms were obtained from GOTERM_BP_DIRECT, and Kyoto Encyclopedia of Genes and Genomes (KEGG) pathways were obtained from KEGG_PATHWAY. Enriched GO terms and KEGG pathways are collectively referred to here as selected functional categories. Benjamini–Hochberg correction was applied for multiple testing, and functional categories with FDR < 0.05 were considered significantly enriched. Bubble plots were used to visualize selected functional categories included in the biological interpretation, with bubble size representing the number of associated genes and color indicating the corresponding FDR. For the exploratory analysis of genes uniquely differentially expressed in resilient birds, bubble plots displayed selected pathways of biological interest. All visualizations were generated in R v4.5.1 using ggplot2 v4.0.2.

### Microfluidic quantitative PCR assay

A subset of RNA-seq-derived differentially expressed genes (DEGs) related to immune response, cell adhesion, antiviral response, and other biological processes of interest was selected for validation by microfluidic quantitative PCR. These genes were analyzed in whole-blood samples collected from the same resilient and susceptible birds included in the RNA-seq analysis, but now using longitudinal samples collected earlier from the same birds at 8 (resilient, n = 8; susceptible, n = 8) and 24 hpi (resilient, n = 7; susceptible, n = 6). Additionally, our previous study demonstrated that significant differences in viral RNA levels in blood and OP and CL swabs between chickens subsequently classified as resilient or susceptible were already evident at 24 hpi and remained detectable at later time points (Valdez-May et al., 2026). Based on these previously established virological profiles, birds euthanized at 24 hpi were provisionally assigned to resilient or susceptible groups according to viral RNA levels in blood and swab samples (resilient, n = 5; susceptible, n = 5). These birds were included in an exploratory analysis aimed at identifying early gene-expression patterns associated with divergent infection outcomes.

Primer design, validation, and detailed methods of microfluidic quantitative PCR procedures were performed according to previously established methods (Valdez-May et al., 2026). All primers included in the analysis are listed in Supplementary Table 1. Total RNA was reverse-transcribed, preamplified, and analyzed using the 48.48 Dynamic Array and on the Biomark HD system (Fluidigm Corporation, South San Francisco, USA) following the manufacturer’s protocols. Gene expression was normalized using reference genes with the lowest M-values: *OAZ1, RPL4*, and *RPL27A*. Statistical analyses of microfluidic qPCR data were performed in GraphPad Prism v8.3.0. The input dataset consisted of normalized relative expression values for each gene in each individual bird. Group differences were evaluated separately at each time point using Welch’s one-way ANOVA, with infection outcome group as the main grouping variable. *P*-values were adjusted using the Benjamini–Hochberg method to control the false discovery rate (FDR), and adjusted *P*-values ≤ 0.05 were considered significant. Heatmaps were generated in R v4.5.1 using ComplexHeatmap v2.24.1. The input matrix consisted of z-score-transformed normalized gene expression values, with genes represented as rows and individual samples as columns. Samples were grouped by infection outcome and time point, and rows were split into predefined functional gene categories of interest. For visualization purposes, samples generated across different chips and experimental runs were combined. Columns were split by experimental group, and hierarchical clustering was performed independently within each column group. Row clustering was performed within each predefined gene category. Default ComplexHeatmap clustering settings were used, corresponding to Euclidean distance and complete-linkage hierarchical clustering.

### Proteomic analysis for blood samples

Sample preparation*.* For the resilient and susceptible groups, three independent pools of three plasma samples were generated at each time point (8, 24, and 48 hpi). For the control group, three independent pools were generated at a single time point. In total, 21 pools were obtained (Supplementary Table 2). For each pool, 2 mL were processed with the iST-BCT sample preparation kit (PreOmics GmbH, Martinsried, Germany), according to the manufacturer's instructions. The resulting peptides were stored at -80°C for subsequent analysis at the Proteomics service of the Center for Genomic Regulation (CRG, Barcelona, Spain).

Liquid chromatography–tandem mass spectrometry (LC–MS/MS). Samples were analyzed using an Orbitrap Eclipse mass spectrometer (Thermo Fisher Scientific) coupled to an EASY-nLC 1200 (Thermo Fisher Scientific). Peptides were separated by reversed-phase chromatography using a 50 cm x 75 μm column packed with 2 μm C18 particles (Thermo Fisher Scientific, cat # ES903). Chromatographic gradients started at 95% buffer A (0.1% formic acid in water) and 5% buffer B (0.1% formic acid in 80% acetonitrile) with a flow rate of 300 nL/min and gradually increased to 25% buffer B and 75% buffer A in 105 min and then to 40% buffer B and 60% buffer A in 15 min. After each analysis, the column was washed for 10 min with 100% buffer B.

The mass spectrometer was operated in positive ion mode using data-independent acquisition (DIA) mode. Full MS scans over a mass range of m/z 500-900 with detection in the Orbitrap at a resolution of 120,000. The auto gain control (AGC) was set to 1e6, and a maximum injection time of 246 ms was used. In each cycle of DIA analysis, following each survey scan, 40 consecutive windows of 10 Da each were used to isolate and fragment all precursor ions from 500 to 900 m/z. A normalized collision energy of 28% was used for higher-energy collisional dissociation fragmentation. MS2 scan range was set from 350 to 1850 m/z with detection in the Orbitrap at a resolution of 30,000. The AGC was set to 1E6, and a maximum injection time of 54 ms was used. Instrument performance and carryover were monitored using digested bovine serum albumin between runs, and QCloud was used for longitudinal quality control (Chiva et al., 2018).

Bioinformatics analysis of proteomic data. Acquired spectra were analyzed using a library-free strategy with DIA-NN (Neural networks and interference correction enable deep proteome coverage in high throughput) (v1.9) (Demichev et al., 2020). The data were searched against the Uniprot Reference Proteome for chicken (*Gallus gallus*; Proteome ID UP000000539, UniProt release 2024_05), accessed and downloaded in October 2024 and containing 18,531 protein entries. The protein sequence database was supplemented with common contaminant proteins, and corresponding decoy precursors were generated automatically within the DIA-NN workflow using the “Shuffle sequence” option and default decoy-generation parameters for target–decoy FDR estimation (Beer et al., 2017). For peptide identification, trypsin was chosen as the enzyme, and up to one missed cleavage was allowed. Oxidation of methionine was used as a variable modification, whereas carbamidomethylation on cysteines was set as a fixed modification. False discovery rate (FDR) was set to a maximum of 1% for peptides and 5% for proteins, respectively. Precursor and fragment ion m/z mass ranges were adjusted to 500-900 and 350-1850, respectively. For peptide quantification, match-between-runs was enabled, protein inference was set to “Protein names (from FASTA)” with “relaxed-prot-inf” option, and the quantification strategy was set to “Robust LC (high precision)”. Default settings were used for the other parameters. The DIA-NN protein-group quantification matrix was subsequently used for downstream differential abundance analysis in FragPipe Analyst v1.9 (Hsiao et al., 2024). Protein-group label-free quantification values were used as input. Before differential abundance analysis, contaminant proteins were filtered out, and protein abundance values were converted to log2 scale. Samples were grouped by experimental condition, and pairwise comparisons were performed between control and resilient, control and susceptible, and resilient and susceptible groups. No additional normalization was applied, and missing values were not imputed. Differential abundance analysis was performed using protein-wise linear models with empirical Bayes statistics using the limma package from R/Bioconductor. *P*-values were adjusted for multiple testing using the Benjamini–Hochberg method to control the FDR. Adjusted *P*-value ≤ 0.05 and |log2 fold change| ≥ 1 were used as thresholds to define significantly differentially abundant proteins. The FragPipe Analyst output contained 3,944 reproducibly quantified protein features, which were used for downstream differential abundance analysis. Proteins detected uniquely in one condition were evaluated separately as presence/absence patterns during biological interpretation when fold change and adjusted *P*-values could not be calculated.

For exploratory functional analysis of differentially abundant proteins, DAVID Bioinformatics Resources v6.8 was used following the same settings described above for the RNA-seq enrichment analysis, including the reference species, annotation categories, multiple-testing correction, and visualization strategy. Protein lists were uploaded as gene symbols and analyzed using *Gallus gallus* as the reference species. The background gene list consisted of all gene symbols corresponding to proteins detected and retained for downstream differential abundance analysis after DIA-NN processing and FragPipe Analyst filtering. Differentially abundant proteins identified in HPAIV-susceptible chickens were selected using adjusted *P*-value ≤ 0.05 and |log2 fold change| ≥ 1. Proteins with higher and lower abundance were analyzed separately within each group. GO Biological Process terms were obtained from GOTERM_BP_DIRECT, and KEGG pathways were obtained from KEGG_PATHWAY. FDR values were reported for each functional category, and categories with FDR < 0.05 were considered significantly enriched. Bubble plots were used to display selected functional categories of biological interest, including both significantly enriched categories and non-significant categories considered relevant for interpretation. All visualizations were generated in R v4.5.1 using ggplot2 v4.0.2.

Transcriptome–proteome correlation analysis. To evaluate the relationship between transcriptomic and proteomic changes, RNA-seq and proteomic differential-expression results were integrated at the gene-symbol level. Because RNA-seq data were available only at 48 hpi, transcriptome–proteome integration was performed as an exploratory analysis. Transcriptomic log2 fold-change values obtained at 48 hpi were compared with proteomic log2 fold-change values measured at 8, 24, and 48 hpi to assess whether protein-level changes detected during early infection were associated with the systemic transcriptional response observed at 48 hpi. Therefore, comparisons involving proteomic data from 8 and 24 hpi were interpreted cautiously, as they did not represent matched transcriptome–proteome measurements collected at the same time point.

For each infection-outcome group, mRNA log2 fold-change values were compared with protein log2 fold-change values derived from the corresponding comparison against controls. Spearman’s rank correlation was calculated using the stats package in R v4.5.1. For each comparison, the number of matched gene–protein pairs, Spearman’s rho, and the corresponding *P*-value were reported. Concordance between transcriptomic and proteomic responses was evaluated based on the direction of fold change: gene-protein pairs were classified as up-up when both mRNA and protein log2 fold-change values were greater than zero, down-down when both values were less than zero, and discordant when the signs differed. The number and percentage of concordantly regulated gene–protein pairs were calculated for each comparison.

All transcriptome–proteome integration analyses were performed in R v4.5.1. Data manipulation was performed using dplyr v1.2.1, and scatterplots were generated using ggplot2 v4.0.2 and ggrepel. Each point represented a matched gene–protein pair. A linear regression line was included only to facilitate visualization of the overall trend, whereas statistical association was assessed using Spearman’s correlation. Representative concordant genes were labeled based on the highest combined absolute transcriptomic and proteomic fold-change magnitude.

Enzyme-linked Immunosorbent Assay (ELISA). Proteomic analysis identified differentially abundant proteins at 48 hpi in resilient birds. However, due to commercial kit availability constraints, only ADP-ribosylation factor 4 (ARF4) was selected for further validation using the commercial ARF4 ELISA kit (BlueGene Biotech), following the manufacturer's protocol. Absorbance was measured at 450 nm using a PowerWave XS reader (BioTek).

## Results

### Transcriptomic profiles differ between clinically defined outcome groups

Among the HPAIV-infected birds, nine died during the study, three developed severe disease (score 3), and one bird exhibited mild clinical signs (score 1). All 13 birds tested positive by IHC and had histopathological lesions consistent with HPAIV infection, including inflammation and necrosis primarily in the spleen, brain, lungs, and heart. In addition, viral RNA was detected in the available OP and CL swab samples collected from these birds at 48 and/or 72 hpi (Ct < 35) (Supplementary Fig. 1). Based on the predefined classification criteria, these 13 birds were classified as susceptible. One additional bird, euthanized at 72 hpi in the absence of overt clinical signs and histopathological lesions associated with HPAIV, tested positive by IHC, and had detectable viral RNA in swab samples. Because this bird did not fully meet the predefined criteria for susceptibility but still showed clear evidence of HPAIV infection, it was classified as an indeterminate infected case and excluded from further comparative analyses. The remaining 25 infected birds survived without severe clinical disease and lacked histopathological and IHC evidence of HPAIV infection, meeting the predefined criteria for classification as resilient. Differences in viral RNA loads between resilient and susceptible chickens were already evident at 24 hpi and persisted at 48 and 72 hpi, as previously described (Valdez-May et al., 2026).

**Fig. 1:**
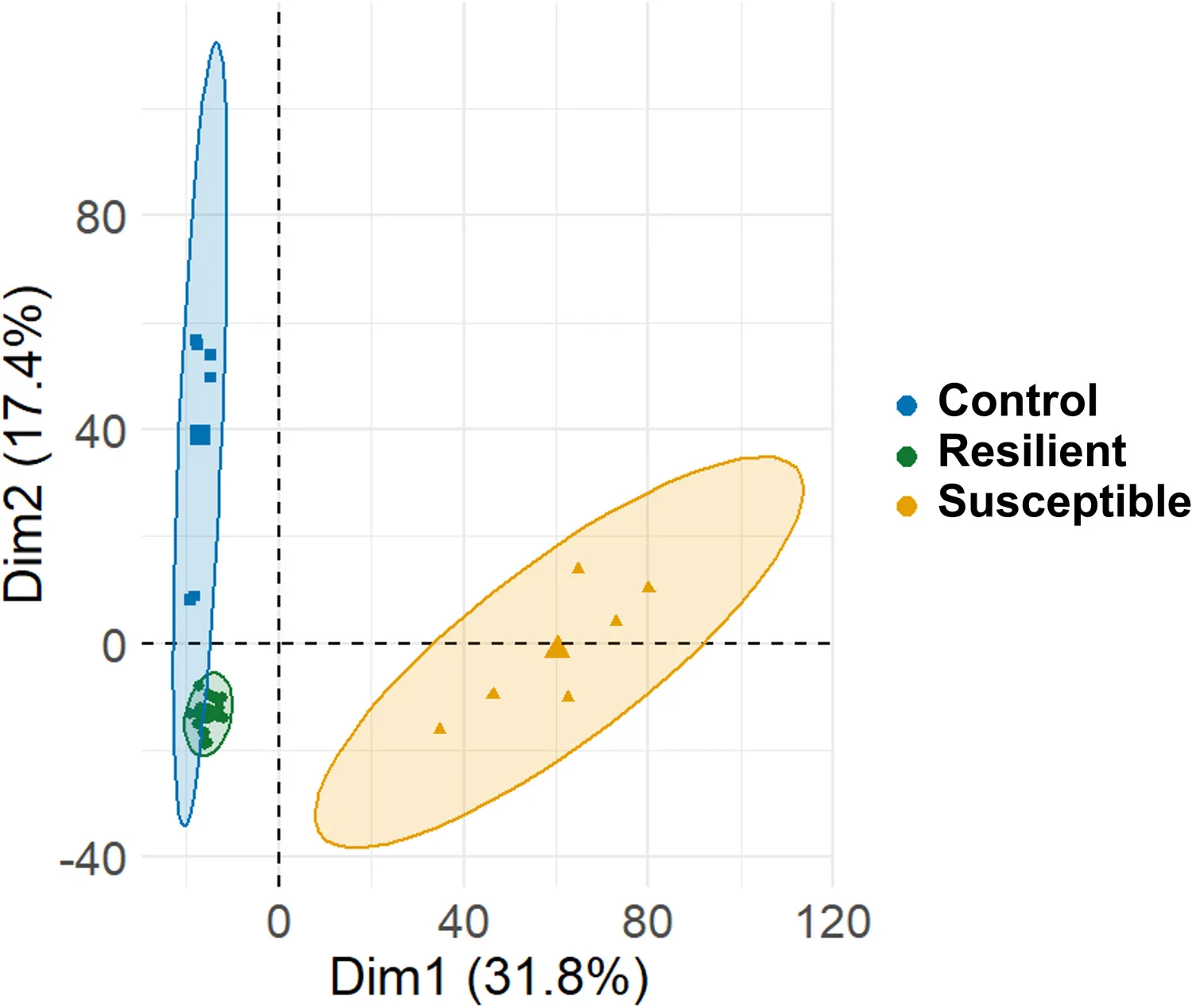
Principal component analysis (PCA) of gene expression data of RNA from whole blood samples from controls (0 hpi), HPAIV-resilient (48 hpi), and susceptible (48 hpi) chickens. Each point represents a sample, color-coded according to the experimental group. The ellipses represent the 95% confidence intervals for each group.

RNA-seq analysis was performed on whole-blood samples collected at 48 hpi from 17 resilient, six susceptible chickens, and on samples collected at 0 hpi from six control chickens. Of the 13 birds classified as susceptible, seven died before the 48-hpi sampling time point and therefore could not be included in the RNA-seq analysis, leaving six susceptible samples available for RNA-seq. Among control and resilient birds, additional samples were excluded because they were unavailable, had insufficient volume, failed to meet predefined RNA quality requirements, or were identified as transcriptomic quality-control outliers. Resilient and susceptible birds were classified based on the clinical, pathological, and virological criteria described above. PCA of the transcriptomic data showed clear separation among the three experimental groups, with control, resilient, and susceptible birds forming distinct transcriptomic clusters (Fig. 1). Furthermore, the PCA plot revealed greater dispersion in gene expression among susceptible birds compared to resilient birds (Fig. 1). Resilient birds formed a compact cluster within the 95% confidence ellipse, clearly separated from controls and susceptible group, indicating a relatively homogeneous systemic transcriptional response after HPAIV challenge, even though viral RNA levels were low in some birds (Fig. 1).

### Major transcriptomic changes are found in both resilient and susceptible chickens despite clear differences in viral counts

**Fig. 2:**
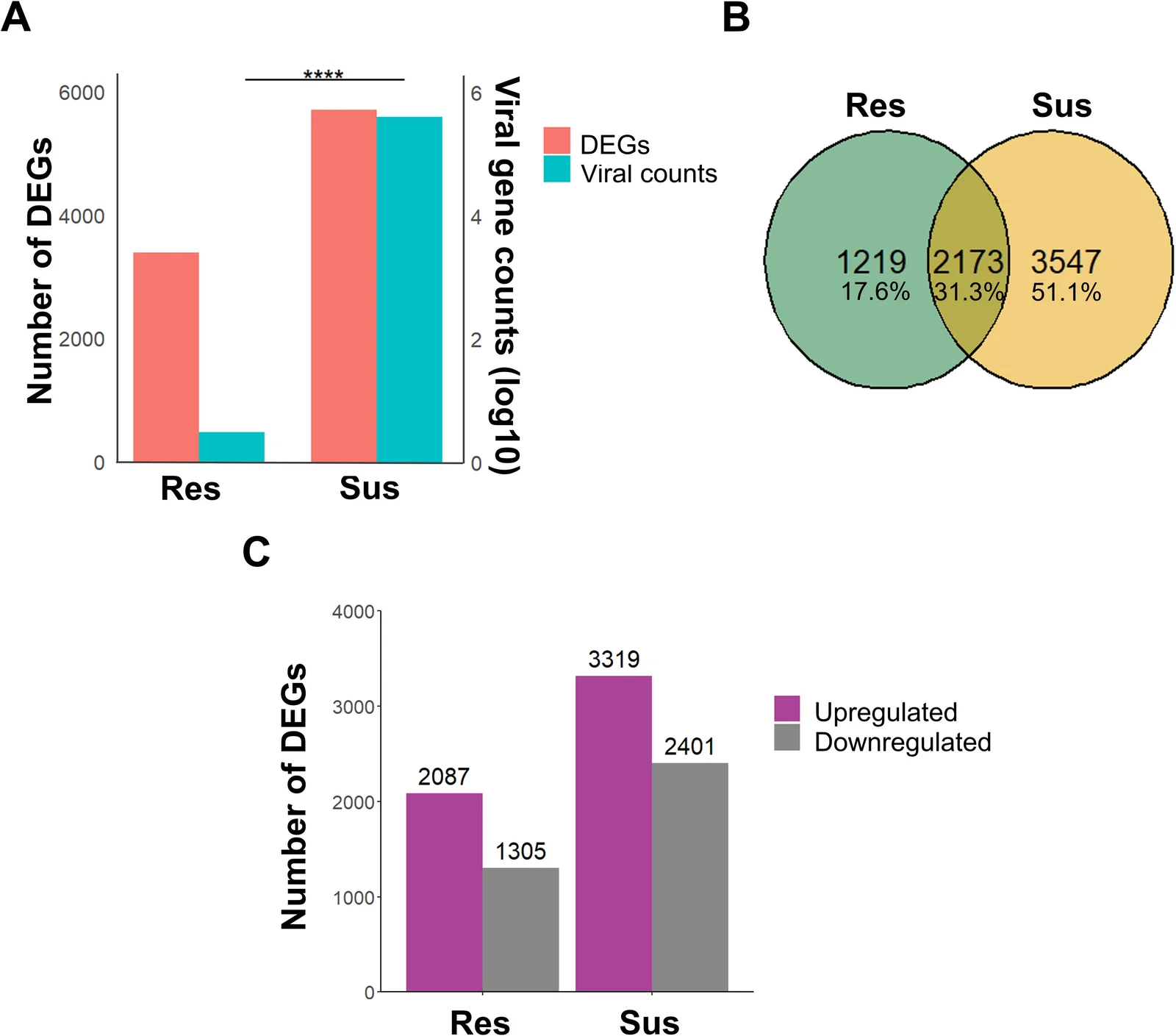
Whole-blood transcriptomic analysis in HPAIV-resilient and HPAIV-susceptible chickens at 48 hpi. (A) Susceptible chickens exhibited significantly more differentially expressed genes (DEGs; salmon) and viral gene counts (turquoise) than resilient chickens. Wilcoxon test ****p ≤ 0.0001. (B) Venn diagram shows overlapping and unique DEGs between groups. (C) The total number of upregulated and downregulated DEGs in each group.

The transcriptomic analysis identified a substantial number of DEGs in both challenged groups, despite significant differences in log10-transformed RNA-seq-derived viral counts between susceptible (5.60) and resilient (0.47) birds (Fig. 2A). Susceptible chickens exhibited more unique DEGs (n = 3,547), representing 51% of the total DEGs identified, than resilient chickens (n = 1,219). Notably, 2,173 DEGs (31%) were shared between both groups, indicating a degree of overlap in the transcriptional response to H7N1 HPAIV infection during the early stages of infection (Fig. 2B). Most DEGs were upregulated in both groups (Fig. 2C).

To further comprehend the biological processes of the identified DEGs at 48 hpi, we performed functional enrichment analysis of DEGs from resilient and susceptible groups. These analyses revealed enrichment of multiple pathways and biological processes related to immune function and host responses to infection. The number of input DEGs, DAVID-mapped genes, and mapped genes annotated in GOTERM_BP_DIRECT and KEGG_PATHWAY for each DEG list are summarized in Supplementary Table 3.

Functional enrichment analysis of DEGs showed that susceptible birds were enriched for antiviral and innate immune response pathways, including Influenza A, RIG-I-like receptor, Toll-like receptor, and innate immune response categories (Supplementary Fig. 2A). In contrast, resilient birds showed enrichment of pathways related to cell adhesion, migration, cytokine signaling, integrin signaling, MAPK signaling, and B cell receptor signaling (Supplementary Fig. 2B). Notably, susceptible chickens displayed upregulation of several immune-related genes compared to both resilient chickens and controls, such as genes involved in apoptosis (e.g., *CASP3, CASP8*), interferon responses (e.g., *IFIT5, MX1*), and pathogen recognition (e.g., *TLR3*) (Supplementary Fig. 3). In contrast, resilient chickens exhibited a more moderate expression profile, with many genes showing lower expression levels than those in the susceptible group (Supplementary Fig. 3). Genes contributing to each enriched functional category are listed in Supplementary Table 4.

### Integrin-mediated, MAPK, and immune responses are linked to resilience to HPAIV infection in chickens

Upon closer examination of the Venn diagram (Fig. 2B), we focused on the functional categories among DEGs identified uniquely in resilient chickens. Upregulated uniquely resilient DEGs were associated with immune and adhesion-related categories, including “adaptive immune response” (GO:0002250), “B cell receptor signaling pathway” (GO:0050853), “T cell costimulation” (GO:0031295), “cell adhesion molecule interaction” (gga04514), “integrin-mediated signaling pathway” (GO:0007229), and “cytokine-cytokine receptor interaction” (gga04060) (Supplementary Fig. 4A). Downregulated uniquely resilient DEGs were associated with selected cellular processes, including “protein transport” (GO:0015031), “regulation of TOR signaling” (GO:0032006), and “alternative mRNA splicing, via spliceosome” (GO:0000380) (Supplementary Fig. 4B). Genes contributing to each functional category are listed in Supplementary Table 4.

**Fig. 3:**
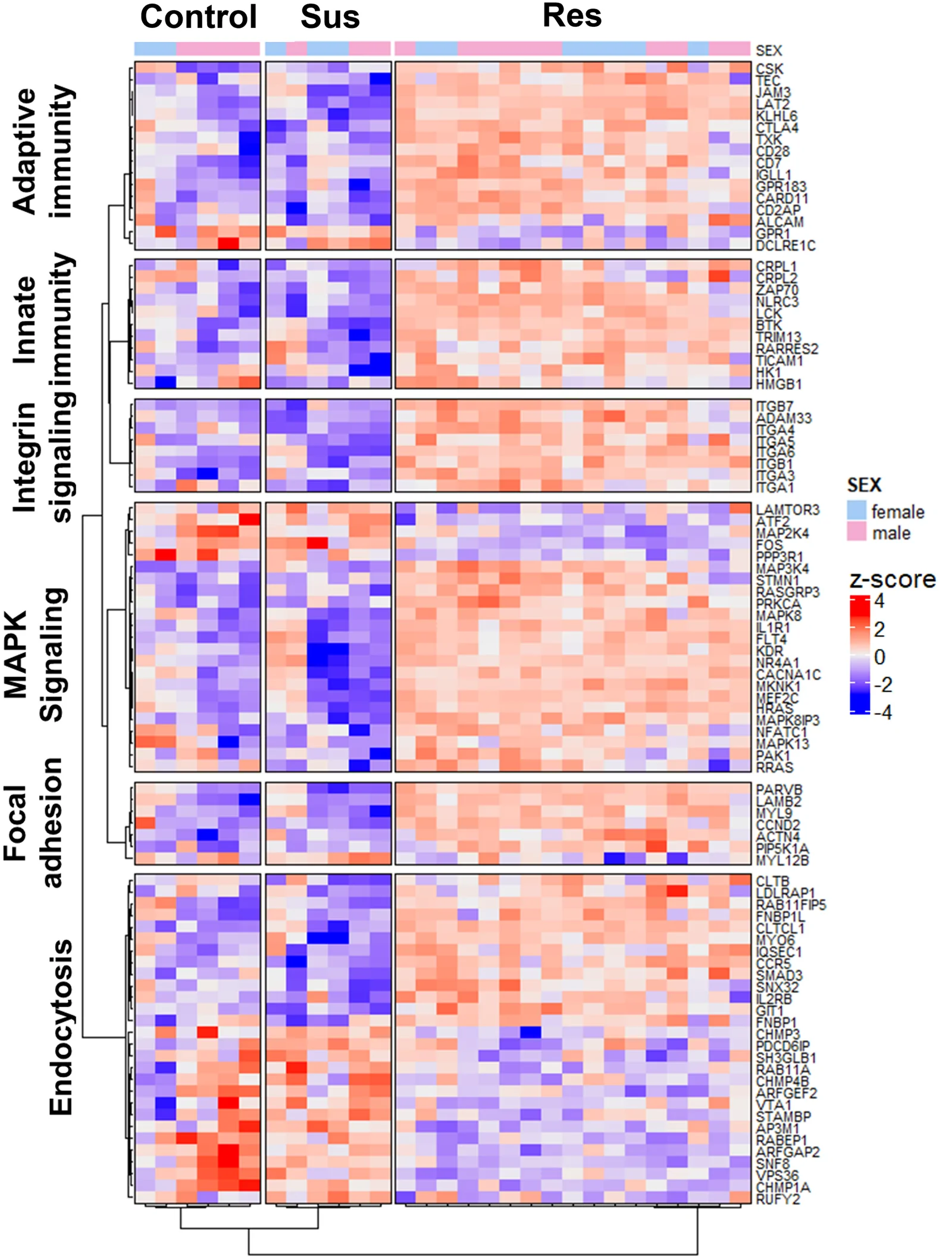
Whole-blood transcriptomic signatures at 48 hpi in HPAIV-resilient, HPAIV-susceptible, and control chickens. The heatmap shows normalized RNA-seq expression of DEGs uniquely identified in resilient chickens. Rows and columns were hierarchically clustered using the R package ComplexHeatmap.

**Fig. 4:**
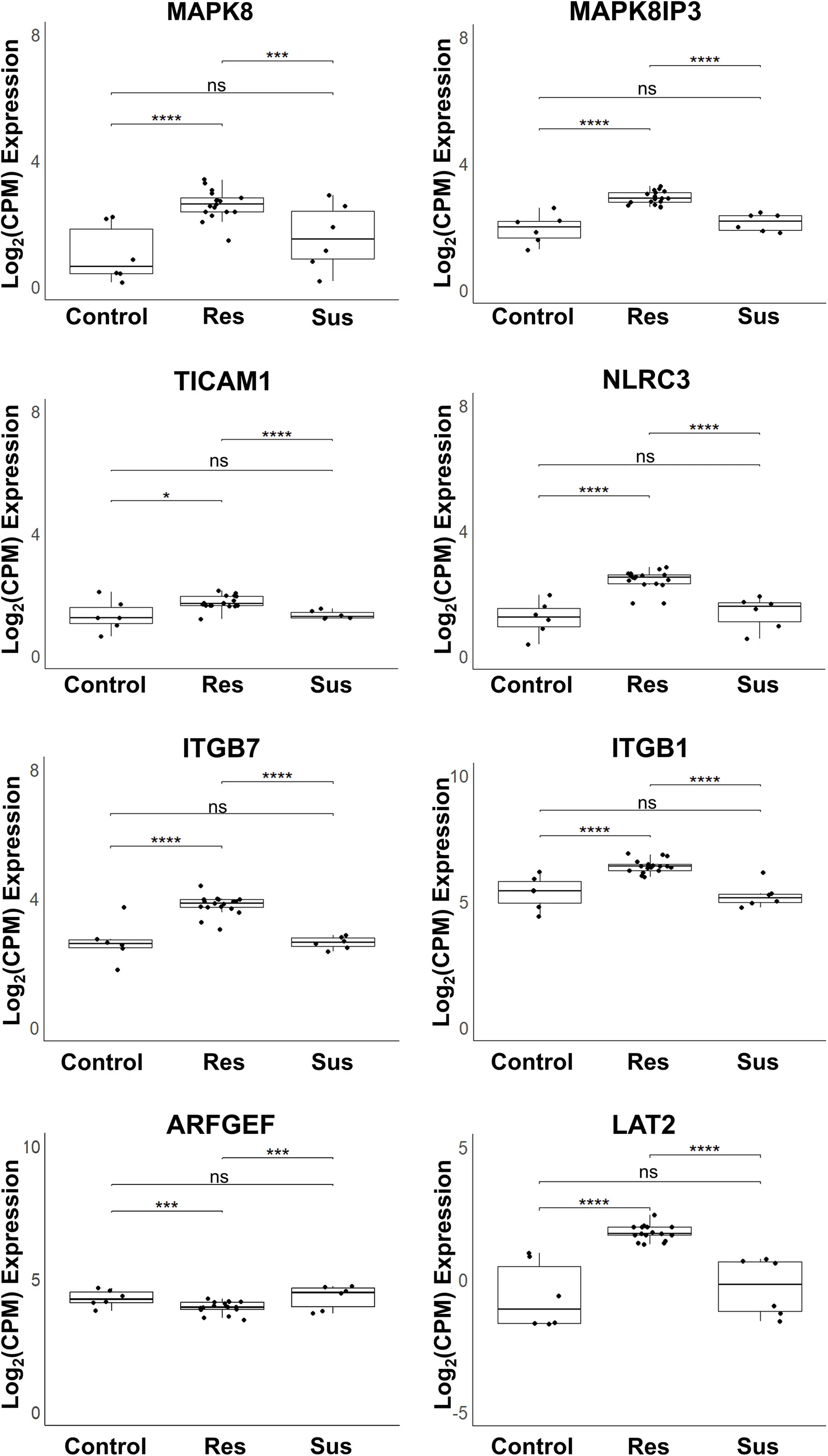
Expression levels of representative genes uniquely identified in HPAIV-resilient chickens at 48 hpi. Boxplots show RNA-seq-derived expression levels (log₂[CPM]) of selected genes. Adjusted *p*-values: ns, not significant; *p* ≤ 0.05 (*), *p* ≤ 0.01 (**), *p* ≤ 0.001 (***), *p* ≤ 0.0001 (****).

Consistent with these pathways, the heatmap of resilient-only DEGs showed higher expression of integrin/adhesion and MAPK modules (e.g., *ITGB1, ITGA5, MAP2K4, ATF2*), together with increased innate (e.g., *TICAM1, NLRC3*) and adaptive (e.g., *CD28, CTLA4, CARD11*) immune signals. In contrast, genes linked to endocytosis displayed mixed expression, with some downregulated (*ARFGAP2, SNF8*) and others upregulated (*CLTCL1, CCR5*) (Fig. 3). Representative genes that were significantly different between resilient birds and both control and susceptible groups include *MAPK13, MAPK8* (MAPK signaling), *TICAM1, NLRC3* (innate antiviral), *ITGB7, ITGB1* (integrin signaling), and *LAT2* (adaptive immunity), whereas *ARFGEF2* (endocytosis/vesicular transport) was downregulated (Fig. 4).

### Gene expression differences between resilient and susceptible chickens are identified before the onset of clinical signs and mortality

**Fig. 5:**
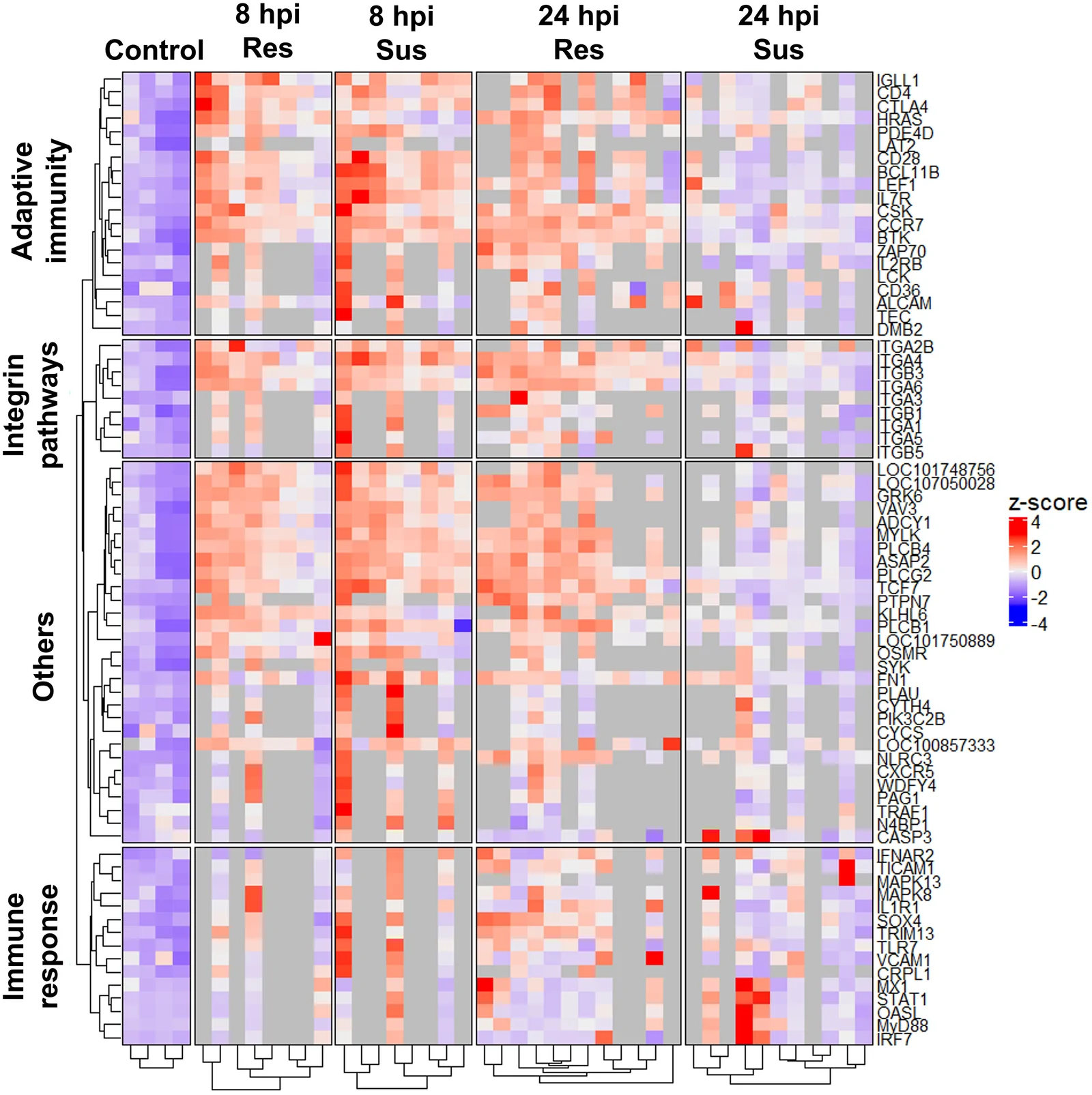
Whole blood transcriptomic signatures at 8 and 24 hpi in HPAIV-resilient, HPAIV-susceptible, and control groups. The heatmap shows z-score-transformed normalized expression values obtained by microfluidic quantitative PCR. Rows and columns were clustered as implemented in the R package ComplexHeatmap.

To validate RNA-seq results, genes identified in resilient chickens at 48 hpi were analyzed retrospectively at 8 and 24 hpi using microfluidic quantitative PCR. At 8 hpi, significant differences in gene expression were observed between HPAIV-infected chickens and the controls, whereas differences between resilient and susceptible chickens were limited to a small number of genes, including *ITGA1, N4BP1*, and *IFNAR2* (Fig. 5). In contrast, at 24 hpi, 17 genes were differentially expressed between HPAIV-resilient and HPAIV-susceptible chickens, and when compared to the control group (Supplementary Fig. 5). Genes involved in the main pathways identified in resilient chickens are listed in Supplementary Table 5.

### Limited proteomic changes are observed at early stages of infection

**Fig. 6:**
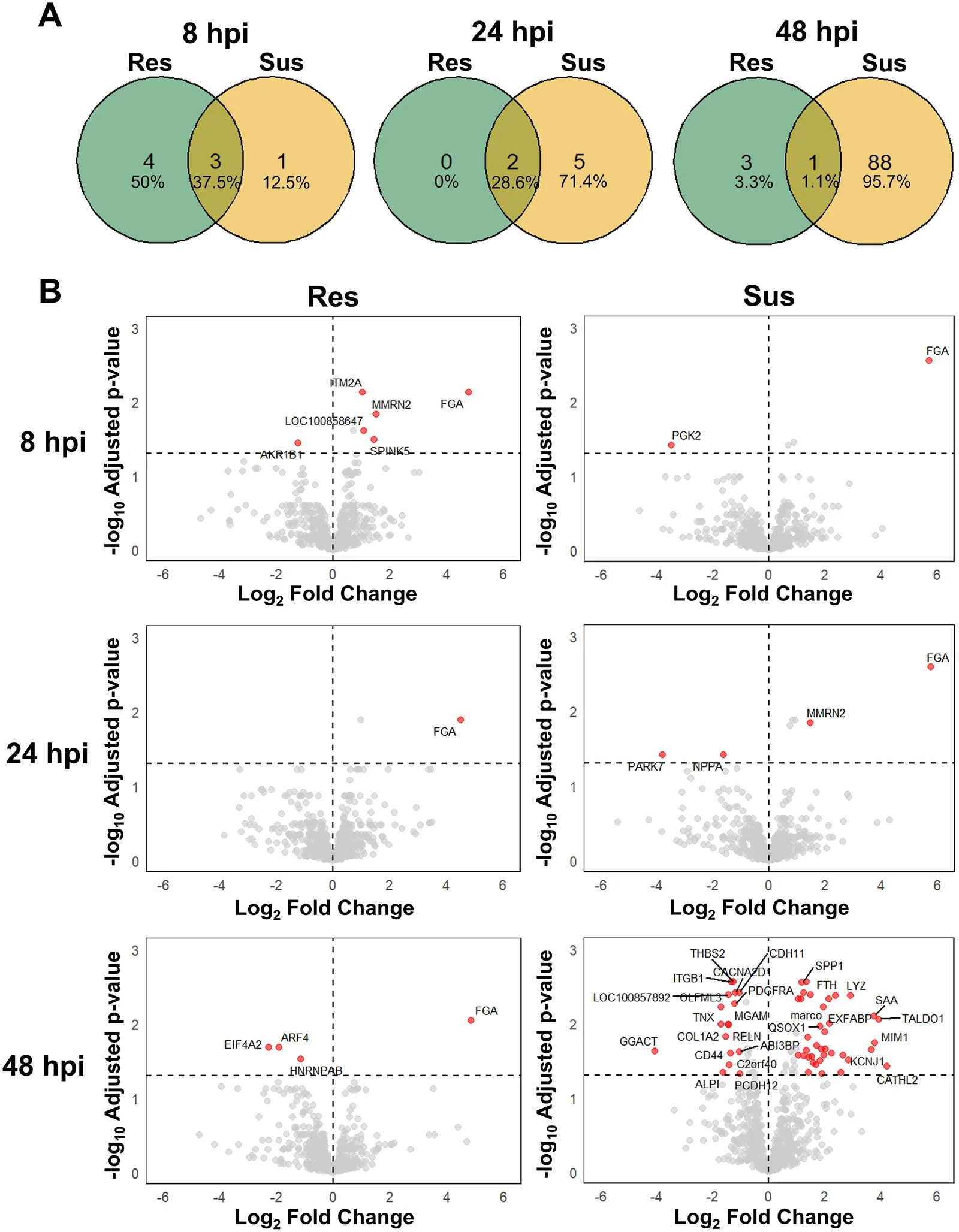
Differentially abundant proteins in plasma samples collected at 8, 24, and 48 hpi from HPAIV-resilient and HPAIV-susceptible chickens compared to controls. Venn diagrams (A) show the number of shared and unique proteins in each group and time point. The Volcano plots (B) display differential protein abundance relative to controls. Each grey dot represents a detected protein, while red dots indicate significantly different proteins (adjusted *p*-value < 0.05 and |log2FC| > 1).

Proteomic results showed few changes at 8 hpi and 24 hpi in plasma samples of both resilient and susceptible chickens compared to controls (Fig. 6A). Few proteins with significantly different abundances between resilient chickens and controls were identified at 8 hpi, including AKR1B1, which showed decreased abundance, and ITM2A, SPINK5, MMRN2, ITM2, and LOC100858647, which showed increased abundance (Fig. 6B). However, none of these proteins showed differential abundance when compared to 8 hpi plasma samples from susceptible birds. By 48 hpi, proteins such as EIF4A2, ARF4, and HNRNPAB were significantly less abundant in resilient chickens compared to controls and susceptible birds (Fig. 6B). On the contrary, HPAIV-susceptible chickens had several differentially abundant proteins compared to controls (Fig. 6B) with increased abundance proteins related to immune defense pathways, including “Acute-phase response” (GO:0006953) and “Complement activation” (GO:0006956) (Supplementary Fig. 6A). In contrast, the downregulated proteins were associated with cellular adhesion and matrix interactions, such as “Focal adhesion” (gga04510) and “Cell adhesion” (GO:0007155) (Supplementary Fig. 6B).

Additionally, an integrative analysis was performed to assess the relationship between whole-blood mRNA levels and plasma protein expression at 8, 24, and 48 hpi (Supplementary Fig. 7). The results suggest a weak but statistically significant association between transcriptomic and proteomic changes in HPAIV-resilient chickens at 48 hpi (ρ=0.17, *p*=1.4e^-03^). Among matched pairs, 21.6% were concordantly upregulated, and 29.3% were concordantly downregulated. In contrast, the association was weaker and not statistically significant in susceptible chickens at 48 hpi (ρ=0.09, *p*=7.7e^-02^), with a higher proportion of proteins (38.5%) showing concordant upregulation (Supplementary Fig. 7).

### Lower ARF4 protein abundance is associated with resilience to HPAIV infection in chickens

**Fig. 7:**
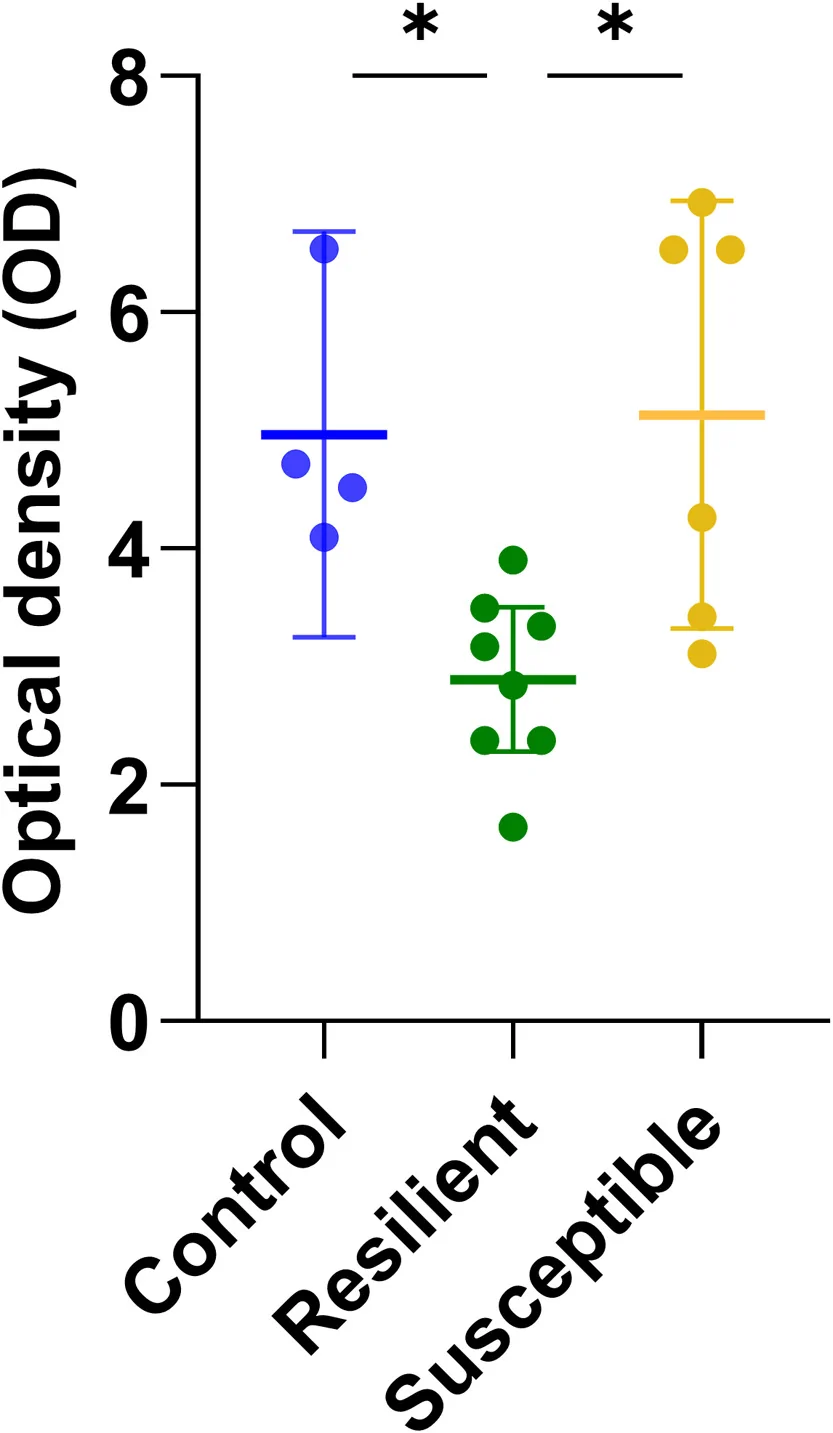
Levels of ARF4 protein in plasma samples of HPAIV-resilient, HPAIV-susceptible, and control chickens at 48 hpi by ELISA. Values are shown as mean with 95% CI. Statistical analysis by one-way ANOVA with post-hoc Tukey’s test: *p ≤ 0.05, **p ≤ 0.01, ***p ≤ 0.001, and ****p ≤ 0.0001.

In HPAIV-resilient chickens, the ARF4 protein was significantly less abundant compared to controls at 48 hpi (Fig. 6B). Remarkably, ARF4 abundance was lower in HPAIV-resilient birds but upregulated in susceptible chickens compared to controls. In line with these results, ARF4 protein levels measured by ELISA were significantly lower in HPAIV-resilient chickens compared to controls and susceptible chickens at 48 hpi (Fig. 7).

## Discussion

The HPAIV infection response is a complex and multifactorial process that involves both host and pathogen factors, contributing to different clinical outcomes in infected birds (Blaurock et al., 2022). Although previous studies have contributed to a better understanding of the mechanisms involved, there are still unanswered questions (An et al., 2019; Perlas et al., 2021). In a previous study, we characterized early transcriptomic responses in the nasal turbinates and lungs, identifying key pathways and genes associated with divergent clinical outcomes following H7N1 HPAIV infection. This study provides a comprehensive analysis of gene and protein changes in the blood of chickens following HPAIV infection. This analysis focuses on the transcriptomic and proteomic differences between resilient and susceptible chickens, thereby shedding light on the biological pathways that were associated with this divergent clinical outcome.

The PCA of the transcriptomic profiles showed clustering according to the independently defined clinical outcome groups. However, because resilience and susceptibility were defined using clinical, pathological, and virological criteria, the PCA separation should be interpreted as an exploratory visualization of transcriptomic differences between outcome groups rather than as an independent validation of the classification. The observed clustering likely reflects multiple biological factors associated with disease outcome, including differences in viral burden, systemic dissemination, and disease severity. In addition, susceptible birds showed greater dispersion across principal components, suggesting greater inter-individual heterogeneity, whereas the tighter clustering of resilient birds was consistent with a more uniform early response. This interpretation is descriptive and does not imply causality. Importantly, resilience should be distinguished from complete resistance to infection. Although viral RNA levels were low or undetectable in some resilient birds, their transcriptomic profiles differed from controls, and viral RNA was previously detected in respiratory tissues from resilient chickens (Valdez-May et al., 2026). Thus, the resilient phenotype is more consistent with reduced viral burden, dissemination, and disease severity than with the absence of infection.

At 48 hpi, both resilient and susceptible birds exhibited substantial transcriptomic changes, despite marked differences in viral load. Importantly, resilient birds showed 3,392 DEGs in blood at 48 hpi, compared with only 39 DEGs previously reported in lung at 72 hpi, and 969 DEGs found in nasal turbinate with the same H7N1 strain (Perlas et al., 2021; Valdez-May et al., 2026). This substantial disparity likely reflects both matrix and time point effects: whole blood captures a rapidly evolving systemic immune response and is well suited for large-scale biomarker discovery (Désert et al., 2016). Moreover, as only a minority of protein-coding genes are strictly tissue-specific, with most expressed across multiple tissues (Djebali, 2012), blood transcriptomes can reflect broad host responses. At the same time, blood represents a composite systemic signal and does not directly resolve molecular events occurring in primary target tissues. Therefore, the signatures identified here should be interpreted as systemic correlates of clinical outcome rather than tissue-specific mechanisms. Furthermore, both resilient and susceptible birds showed increased expression of DEGs related to immune-related, inflammatory, and IFN-I response DEGs compared with controls. However, susceptible birds showed a significantly greater number of DEGs involved in the the immune response against HPAIV (Ranaware et al., 2016). These results suggest that resilient chickens exhibit a more coordinated systemic transcriptional response at early stages of infection, potentially compatible with reduced inflammatory dysregulation while maintaining antiviral defense. Notably, DEGs in resilient birds were associated with more functional categories, particularly those related to inflammation, immune response, and viral response, including KEGG pathways such as Influenza A. This supports the interpretation that resilient birds mounted an immune response against AIV infection.

Functional categories of the DEGs identified uniquely in the resilient group highlighted pathways potentially associated with lower viral burden and a favorable outcome. Pathways related to innate immune response (e.g., *TICAM1*), as well as MAPK components (*MKNK1, MAPK13, MAPK8*) and T-cell signaling (*LCK, ZAP70, LAT2*), suggest a coordinated involvement of both innate and adaptive immune systems at early stages (Matsumoto and Seya, 2008; Xing et al., 2010). The MAPK cascade regulates cell proliferation, differentiation, and apoptosis and has previously been linked to mechanisms associated with resilience to AIV infection, suggesting that it may contribute to shaping antiviral and inflammatory responses (Perlas et al., 2021; Vu et al., 2022, Vu et al., 2023b). The integrin-mediated signaling pathway was also identified in resilient birds, including upregulation of *ITGA5, ITGA6*, and *ITGB1*, which may support immune cell migration and the formation of stable immune synapses between T cells and antigen-presenting cells (Ratcliffe et al., 2016). Furthermore, integrins help to maintain epithelial barrier integrity and promote tissue repair, and integrin expression has also been reported following AIV infection (Jolly et al., 2014; Han et al., 2024). In addition, several genes associated with T-cell signaling (e.g., *HRAS, BTK, ZAP70, LCK, LAT2*) were identified in resilient birds, supporting coordinated activation of both innate and adaptive immune responses (Singer et al., 2003), in line with recent evidence linking stronger TCRγδ+CD8α+ T-cell responses to improved early control of AIV infection in more resistant chicken lines (Dai et al., 2025). Overall, these signatures suggest that resilient chickens mount a coordinated early systemic response involving both innate and adaptive immune pathways. This pattern is consistent with a more favorable host response to HPAIV infection, although functional validation will be required to determine whether these pathways play a causal role in limiting viral burden or disease severity ( Vu et al., 2023a).

Consistent with the results on viral shedding and viremia, DEGs were identified between resilient and susceptible birds as early as 8 and 24 hpi, before the onset of clinical signs and mortality at 48 hpi. This early gene expression difference suggests that the host response to HPAIV initiates rapidly and may be associated with subsequent clinical outcome. The upregulation of genes associated with innate and adaptive immune responses, integrin-mediated pathways, and cell adhesion genes in resilient chickens contrasts with their downregulation in susceptible ones, indicating a distinct immune response in resilient birds compared to a more dysregulated response in susceptible chickens. Furthermore, identifying DEGs before the onset of mortality highlights candidate markers that may be useful in future studies aimed at selective breeding or gene-editing strategies to promote protective immune responses in poultry populations. A more comprehensive analysis, including RNA-seq at earlier time points in blood samples, could provide deeper insight into the initial host response to HPAIV before the onset of clinical signs.

The integrative omics analysis revealed a weak positive correlation between mRNA and protein expression levels. This may be partly explained by the fact that transcriptomic approaches offer greater sensitivity than proteomic analysis (Ponomarenko et al., 2023). Moreover, while transcriptomic data can offer important insights into protein abundance, post-transcriptional and post-translational regulatory mechanisms influence the final protein abundance (Zhong et al., 2023). These findings are consistent with previous studies in chickens, where the mRNA-protein correlation has been reported to range from low to moderate (r ≈ 0.14–0.6), highlighting the complexity of protein-level regulation (Liu et al., 2020; Wang et al., 2025). These factors may partially explain the limited protein detection observed in resilient chickens across all time points, particularly the marked reduction at 48 hpi relative to mRNA expression levels. Among the proteins of interest, ARF4, which is significantly lower in abundance in resilient chickens at 48 hpi, emerged as a candidate associated with the resilient phenotype. In human cell models, the loss of ARF4 prevents influenza A virus from egressing via Golgi trafficking (Li et al., 2025); whether a similar mechanism operates in chicken blood cells remains to be tested. Likewise, lower EIF4A2 abundance is consistent with reports suggesting that reduced EIF4A2 activity can limit RNA virus replication (Song et al., 2019), but EIF4A2 is a core translation factor, and effects may be context-dependent.

An additional consideration is that all birds were vaccinated at hatch with a live attenuated infectious bronchitis virus vaccine, reflecting standard commercial practice. Although vaccination was applied uniformly across all individuals, prior immune priming may have influenced baseline or systemic immune responses, particularly those related to adaptive immunity. Therefore, some of the transcriptional signatures observed in blood may partially reflect vaccine-associated immune modulation in addition to responses elicited by HPAIV infection.

To summarize, this study provides insight into systemic molecular signatures associated with a favorable outcome following HPAIV infection in chickens. Key findings identify MAPK, integrin, and immune-signaling modules as early systemic correlates of resilience, while EIF4A2 and ARF4 are identified as promising proteins for further investigation. However, whole-blood profiling captures systemic signatures but may conflate regulatory changes with shifts in circulating cell populations; without cytometric counts or deconvolution data, cellular sources cannot be precisely resolved. In addition, the modest sample size limits the statistical power to detect subtle effects and inter-individual variability. Cross-omics integration was also constrained by proteome depth, particularly at 48 hpi. Future work should (i) adjust for blood cell composition, and (ii) perform perturbations in avian cells (e.g., CRISPR/siRNA knockdown, pharmacologic inhibition) to test causality. In conclusion, our findings define early systemic responses associated with resilient outcomes following HPAIV infection and provide a foundation for future studies aimed at validating candidate markers and exploring strategies to enhance resilience in chickens.

## Data availability statement

The mass spectrometry raw spectra generated in this study have been deposited in the PRIDE repository with the dataset identifier PXD067000. The raw RNA-seq sequencing reads generated in this study have been deposited in the NCBI SRA under the BioProject accession number PRJNA1346088.

## Ethics statement

The experimental protocol was approved by the Institutional Animal Care and Use Committee and the Catalan Government under permit number CEA-OH/11722/2.

## Author Contributions

KB and NM conceived the project and acquired funding. The experiment was designed by MJV-M, KB, and NM. Experimental work was performed by MJV-M, MN, KB, and NM, and samples were collected and processed by MJV-M, AP, MN, RV, and AM. MJV-M, MD, AE-C, and JA conducted the RNA-seq analysis. MJV-M, MP, and SP-P performed the microfluidic quantitative PCR analyses. MJV-M, JA, and KB prepared the figures. MJV-M drafted the manuscript, and KB and NM contributed to manuscript revision and editing. All authors reviewed and approved the final manuscript.

## Funding

This work was funded by the 10.13039/501100004837Spanish Ministry of Science and Innovation within the coordinated project PID2020-114060RR-C33-INFLUOMA, “Unravelling the molecular mechanisms of avian influenza virus infection outcome in the avian host by using a multi-omics approach”. MJV-M is supported by a doctoral scholarship from the Mexican Secretaría de Ciencia, Humanidades, Tecnología e Innovación (Secihti, CVU 1070914). KB is funded by the Ministry of Economy and Competitiveness, through the Ramón y Cajal Program (Grant RYC2021-033472-I).

## Disclosures

The authors declare that they have no known competing financial interests or personal relationships that could have appeared to influence the work reported in this paper.

## Appendix Supplementary materials

Image, application 1

Supplementary Table 2. **Summary of analyses performed on whole blood samples collected from controls, HPAIV-resilient, and HPAIV-susceptible chickens.** Samples collected at 48 hours post-inoculation (hpi) were analyzed using RNA-seq, while samples from 8 and 24 hpi were quantified using microfluidic quantitative PCR. Because the onset of mortality was at 48 hpi, the number of samples analyzed from susceptible birds for this time point was limited, as not all susceptible birds could be sampled before death. Additionally, only samples with high RNA quality were selected for further analysis. Furthermore, proteomic analysis was performed using pooled samples at 0, 8, 24, and 48 hpi. Different colors represent the pooled samples for each time point. Asterisks (*) represent outliers from the susceptible group at 48 hpi that were discarded from further analysis. Image, application 2

Image, application 3

Image, application 4

Image, application 5

Image, image 6 dummy alt text

Supplementary Figure 2. **Functional enrichment analysis of significantly enriched Gene Ontology (GO) Biological Process terms and KEGG pathways identified in whole blood of HPAIV-susceptible (A) and HPAIV-resilient (B) chickens collected at 48 hours post-inoculation (hpi).** Dot color indicates enrichment significance, expressed as -log10 of the false discovery rate (FDR), whereas dot size represents the number of differentially expressed genes (DEGs) associated with each functional category. Only categories meeting the significance threshold of FDR < 0.05 are shown. Image, image 7 dummy alt text

Supplementary Figure 3. **Whole blood transcriptomic signatures at 48 hours post-inoculation (hpi) in HPAIV-resilient, HPAIV-susceptible, and control groups.** The heatmap displays the normalized RNA-seq expression levels of selected differentially expressed genes (DEGs) across samples. The heatmap was generated using R package *ComplexHeatmap*. Image, image 8 dummy alt text

Image, image 9 dummy alt text

Supplementary Figure 5. **Normalized expression levels of genes in blood samples collected at 24 hours post-inoculation (hpi) from control, HPAIV-resilient (Res), and HPAIV-susceptible (Sus) chickens.** These genes exhibited significant differences between resilient and susceptible birds at 24 hpi. The y-axis represents the average normalized expression levels for the control group (black), HPAIV-resilient chickens (light gray), and HPAIV-susceptible chickens (dark gray). The mean and standard deviation are represented. Statistical significance was determined using Welch's one-way ANOVA; **p* ≤ 0.05, ***p* ≤ 0.01, ****p* ≤ 0.001, and *****p* ≤ 0.0001. Image, image 10 dummy alt text

Supplementary Figure 6. **Exploratory functional analysis of Gene Ontology (GO) Biological Process terms associated with differentially abundant proteins in plasma samples from HPAIV-susceptible chickens at 48 hours post-inoculation (hpi).** Panels show proteins with increased abundance (A) and decreased abundance (B). Dot color indicates enrichment significance, expressed as -log_10_(FDR), whereas dot size corresponds to the number of differentially abundant proteins associated with each term. Image, image 11 dummy alt text

Supplementary Figure 7. **Exploratory integrative transcriptomic and proteomic analysis.** Correlation between transcriptomic (48 hours post-inoculation (hpi)) and proteomic responses (8, 24, and 48 hpi) in HPAIV-resilient and HPAIV-susceptible chickens. Scatterplots compare transcriptomic log_2_ fold changes (log_2_FC; x-axis) with proteomic log_2_ fold changes (log_2_FC; y-axis). Each dot represents a matched gene-protein pair. The solid line represents the fitted regression line, and Spearman's correlation coefficients are shown to assess the association between transcriptomic and proteomic changes. Points are colored according to concordant direction of change (red, increased-increased; blue, decreased-decreased), whereas discordant pairs are shown in gray. The number and percentage of concordant pairs are reported at the top of each plot. Selected representative gene-protein pairs are labeled. Image, image 12 dummy alt text

## Footnotes

Supplementary material associated with this article can be found, in the online version, at doi:10.1016/j.psj.2026.107527.

## Footnotes

1 International Committee on Taxonomy of Viruses (ICTV). 2026. ICTV Taxonomy. Accessed Aug. 7, 2026. https://ictv.global/taxonomy

2 World Organisation for Animal Health (WOAH). 2024. Manual of Diagnostic Tests and Vaccines for Terrestrial Animals. 13th ed. World Organisation for Animal Health, Paris, France. Accessed Aug. 7, 2026. https://www.woah.org/fileadmin/Home/eng/Health_standards/tahm/A_summry.htm
